# A tension spline fitted numerical scheme for singularly perturbed reaction-diffusion problem with negative shift

**DOI:** 10.1186/s13104-023-06361-8

**Published:** 2023-06-22

**Authors:** Ababi Hailu Ejere, Tekle Gemechu Dinka, Mesfin Mekuria Woldaregay, Gemechis File Duressa

**Affiliations:** 1 Department of Applied Mathematics, Adama Science and Technology University, Adama, Ethiopia; 2 Department of Mathematics, Jimma University, Jimma, Ethiopia

**Keywords:** Singularly perturbed problem, Tension spline method, Boundary layers, Uniform convergence, Primary 65M06, Secondary 65M12, 65M15, 65M22

## Abstract

**Objective:**

The paper is focused on developing and analyzing a uniformly convergent numerical scheme for a singularly perturbed reaction-diffusion problem with a negative shift. The solution of such problem exhibits strong boundary layers at the two ends of the domain due to the influence of the perturbation parameter, and the term with negative shift causes interior layer. The rapidly changing behavior of the solution in the layers brings significant difficulties in solving the problem analytically. We have treated the problem by proposing a numerical scheme using the implicit Euler method in the temporal direction and a fitted tension spline method in the spatial direction with uniform meshes.

**Result:**

Stability and uniform error estimates are investigated for the developed numerical scheme. The theoretical finding is demonstrated by numerical examples. It is obtained that the developed numerical scheme is uniformly convergent of order one in time and order two in space.

## Introduction

In various areas of science and engineering, one may assume that a certain system is governed by a principal cause, which means that the current state is not dependent on the previous state and determined solely by the present one. However, under closer observation, a principal cause is usually an approximation to the real situation and more existent models involve some of the past states of the system. Such systems are governed by delay differential equations. Delay differential equations have recently gained popularity in a variety of fields of study, such as biology, engineering, robotics, and others with different goals and expectations [1].

A singularly perturbed delay reaction-diffusion problem is a differential equation in which the diffusive term is dominated by the reaction term due to the small positive parameter $\varepsilon$ and involves one or more shifting arguments. Such problems arise frequently in the modeling of different physical phenomena. For instance, models in Bio-mathematics [2], problems in optimal control theory [3], neural dynamics and signal transmission [4] and models in the electro-optic bistable devices [5] are some applications modeled using singularly perturbed delay differential equations.

Due to the presence of $\varepsilon$ as a coefficient of the highest order derivative term, the solution of a singularly perturbed delay differential equation varies abruptly involving two boundary layers. The term with large delay gives rise to interior layer. The abruptly changing behaviors of the solution in the layers make it difficult to solve the problem analytically.

Standard numerical methods are unfit to provide acceptable approximations to the solution of singularly perturbed problems due to the presence of layers. So, there is a need of developing uniformly convergent numerical methods to treat such type of problems.

Various research works are available in the literature to address the aforementioned limitations. For instance, Duressa [6] constructed a numerical method for singularly perturbed differential equation involving small delay by introducing a fitting parameter applying the finite difference approximation. Woldaregay and Duressa [7] developed a hybrid finite difference method on uniform meshes for singularly perturbed problem with delay. Chakravarthy et al. [8] treated a singular perturbation problem with delay by formulating a scheme using cubic spline in compression on a uniform mesh. Daba and Duressa [9] solved singularly perturbed problems by formulating a hybrid numerical scheme on a piece-wise uniform spatial meshes. Bansal and Sharma [10] solved singularly perturbed problems involving large delay by formulating a numerical method applying implicit Euler method in time variable and central difference method in space variable with piece-wise uniform meshes. Kumar and Kumari [11] developed numerical schemes for singularly perturbed parabolic reaction-diffusion problem with delay based on the Crank-Nicolson method for the time variable and the central difference approach for the spatial variable with a non-uniform meshes. Ejere et al. [12] proposed a fitted mesh numerical scheme for a singularly perturbed parabolic reaction-diffusion problem with large delay using the weighted average method in the time variable and central difference method in the spatial variable, and obtained that the method is uniformly convergent.

Motivated by the various papers mentioned above, we treated a time dependent singularly perturbed parabolic differential equation with delay in the spatial variable. We handled the influence of the perturbation parameter and the large negative shift by developing a numerical scheme based on the implicit Euler method in the time direction and a fitted tension spline method in the spatial direction on uniform meshes. The stability estimate and the uniform convergence of the proposed numerical scheme are investigated and proved. The validity of the theoretical findings is demonstrated by carrying out numerical experiments. Based on the theoretical and numerical results, we found that the proposed scheme is uniformly convergent.

The remainder of this paper is organized in the following order: In Sect. "Continuous problem", we present the statement of the problem. Section "Numerical Method" deals with the detail numerical description and methods. We present numerical results and discussions to illustrate the theoretical results in Sect. "Numerical experiments, results and discussions". We give the conclusion of this research work in Sect. "Conclusion".

Notation: Throughout this paper, we denote *C* as a generic constant independent of the perturbation parameter and the mesh numbers, which may take different values in different inequalities or equations. For a given function $\upsilon$ on a domain $\Omega$, the maximum norm is defined as $\Vert \upsilon \Vert =\max \limits _{{(x,t)}\in {\bar{\Omega }}}|\upsilon (x,t)|$.

## Continuous problem

We consider the following singularly perturbed delay differential equation on $\Omega =\Omega _{x} \times \Omega _{t}=[0,2]\times [0,T]$.

1 $$\begin{aligned} {\left\{ \begin{array}{ll} { \frac{\partial u(x, t)}{\partial t}-\varepsilon \frac{\partial ^{2} u(x, t)}{\partial x^{2}}+l(x)u(x,t)+m(x)u(x-1, t)=g(x, t)},\\ {u(x,0)=u_{0}(x),\; x\in [0,2]},\\ u(x,t)=\alpha (x,t),\; (x,t) \in \Omega _{L},\\ u(2,t)=\beta (t), \; (2,t) \in \Omega _{R}, \end{array}\right. } \end{aligned}$$

where $0< \varepsilon \ll 1$, $\Omega _{L}=\{(x,t):\; x\in [-1,0]; \; t\in [0, T] \}$ and $\Omega _{R}=\{(2,t): t\in [0, T]\}$ for finite time *T*. The functions *l*(*x*), *m*(*x*), *g*(*x*, *t*), $u_{0}(x)$, $\alpha (x,t)$ and $\beta (t)$ are assumed to be sufficiently smooth, bounded and independent of $\varepsilon$. Moreover, for arbitrary positive constant $\mu$, we assumed that

2 $$\begin{aligned} l(x)+m(x)\ge 2\mu > 0 \; \text {and} \; m(x)<0,\; x \in {\bar{\Omega }}_{x}. \end{aligned}$$

Considering the interval boundary conditions, Eq. (1) can be equivalently written as

3 $$\begin{aligned} L_{\varepsilon }u(x,t) = {\left\{ \begin{array}{ll} \frac{\partial u(x, t)}{\partial t}-\varepsilon \frac{\partial ^{2} u(x, t)}{\partial x^{2}}+l(x)u(x,t)=g(x,t)-m(x){\alpha (x-1, t)}, \\ \qquad (x,t)\in (0,1] \times (0,T], \\ \frac{\partial u(x, t)}{\partial t}-\varepsilon \frac{\partial ^{2} u(x, t)}{\partial x^{2}}+l(x)u(x,t)+m(x)u(x-1, t)=g(x, t), \\ \qquad (x,t)\in (1,2)\times (0,T] \end{array}\right. } \end{aligned}$$

subject to

4 $$\begin{aligned} {\left\{ \begin{array}{ll} u(x,0)=u_{0}(x), \; \forall x \in {\bar{\Omega }}_{x}, \\ u(x,t)=\alpha (x,t), \; (x,t) \in \Omega _{L}, \\ u(2,t)=\beta (t), \; (2,t)\in \Omega _{R}. \end{array}\right. } \end{aligned}$$

If we set $\varepsilon =0$ in the continuous problem, then the reduced problem is given as

5 $$\begin{aligned} L_{\varepsilon } u_{0}(x, t)= {\left\{ \begin{array}{ll} \frac{\partial u_{0}(x, t)}{\partial t}+l(x)u_{0}(x,t)=g(x,t)-m(x){\alpha (x-1,t)}, \\ \; \; \; \; \; \; (x, t)\in (0,1]\times (0,T],\\ \frac{\partial u_{0}(x, t)}{\partial t} +l(x)u_{0}(x,t)+m(x)u_{0}(x-1,t)=g(x,t),\\ \; \; \; \; \; \; (x, t)\in (1,2)\times (0,T] \end{array}\right. } \end{aligned}$$

with the conditions

6 $$\begin{aligned} {\left\{ \begin{array}{ll} u_{0}(x,0)=u_{0}(x), \; x \in {\bar{\Omega }}_{x}, \\ u_{0}(x,t)=\alpha (x,t), \; (x,t) \in \Omega _{L}, \\ u_{0}(2,t)=\beta (t), \; (2,t)\in \Omega _{R}. \end{array}\right. } \end{aligned}$$

From the reduced form in Eq. (5), we observed that $u_{0}(x,t)$ needs not necessarily satisfy the conditions

$$\begin{aligned} u_{0}(0,t)&=\alpha (0,t),\; u_{0}(2,t)=\beta (t),\; u_{0}(1^{+},t)=u_{0}(1^{-},t)\\&\quad \text {and} \;\; {(u_{0})_{x}(1^{+},t)=(u_{0})_{x}(1^{-},t)} \end{aligned}$$

and hence, the solution *u*(*x*, *t*) involves two boundary layers at the ends of [0, 2] and interfacing layers at $x=1$ [13]. Moreover, the initial and boundary data are assumed to satisfy Holder continuity and we impose the compatibility conditions as

7 $$\begin{aligned}&{\left\{ \begin{array}{ll} u_{0}(0,0)=\alpha (0,0), \\ u_{0}(2,0)=\beta (2,0), \end{array}\right. } \end{aligned}$$

8 $$\begin{aligned}&{ {\left\{ \begin{array}{ll} \frac{\partial \alpha (0, 0)}{\partial t}-\varepsilon \frac{\partial ^{2} u_{0}(0, 0)}{\partial x^{2}}+l(0)u_{0}(0, 0)=g(0,0)-m(0)\alpha (-1.0), \\ \frac{\partial \beta (2, 0)}{\partial t} -\varepsilon \frac{\partial ^{2} u_{0}(2, 0)}{\partial x^{2}}+l(2)u_{0}(2, 0)+m(2)u_{0}(1,0)=g(2,0). \end{array}\right. }} \end{aligned}$$

By the above assumptions, it is possible to obtain a unique solution for the considered continuous problem. And by the approaches in [14, 15], we can obtain that

9 $$\begin{aligned} \left| u(x,t)-u_{0}(x)\right| \le Ct,\; (x,t)\in {\bar{\Omega }}. \end{aligned}$$

The solution to Eq. (1) approaches to $u_{0}(x,t)$ for small values of $\varepsilon$. As it is described in [16], we assumed that all the considered data values in Eq. (1) are identically zero, so that the following properties hold.

### Lemma 2.1

The solution *u*(*x*, *t*) of the continuous problem (1) is bounded as $\left| u(x,t)\right| \le C$, $(x,t)\in {\bar{\Omega }}$.

### Proof

From Eq. (9), it follows that $\left| u(x,t)\right| -\left| u_{0}(x)\right| \le Ct$, which implies that

$$\begin{aligned} \left| u(x,t)\right| \le Ct+\left| u_{0}(x)\right| , (x,t)\in {\bar{\Omega }}. \end{aligned}$$

Since $$u_{0}(x)$$ is bounded, fixing *t* in (0, *T*], we obtain $$\left| u(x,t)\right| \le C$$, $$(x,t)\in {\bar{\Omega }}$$$$\square$$.

### Lemma 2.2

(Maximum principle). Let *z*(*x*, *t*) be a continuous function in ${\bar{\Omega }}$. If $z(x,t)\ge 0$, $(x,t)\in \partial \Omega$ and $L_{\varepsilon } z(x,t)\ge 0$, $(x,t) \in \Omega$, then $z(x,t)\ge 0$, $(x,t) \in {\bar{\Omega }}$.

### Proof

Let $({\hat{x}}, {\hat{t}} )\in {\bar{\Omega }}$ and $z({\hat{x}},{\hat{t}})=\min _{{\bar{\Omega }}}z(x,t)$. Assume that $z({\hat{x}},{\hat{t}})< 0$. By the considered hypothesis, $({\hat{x}},{\hat{t}})\notin \partial \Omega$ and by the extreme value theorem, we have $z_{x}({\hat{x}},{\hat{t}})=0$, $z_{xx}({\hat{x}},{\hat{t}})\ge 0$.

**Case 1:** For $0<{\hat{x}}\le 1$, we have

$$\begin{aligned} { L_{\varepsilon ,1} z({\hat{x}},{\hat{t}})=z_{t}-\varepsilon z_{xx}+l({\hat{x}})z({\hat{x}},{\hat{t}})=-\varepsilon z_{xx}({\hat{x}},{\hat{t}})+l({\hat{x}})z({\hat{x}},{\hat{t}})< 0.} \end{aligned}$$

**Case 2:** For $1<{\hat{x}}\le 2$, we have

$$\begin{aligned} { L_{\varepsilon ,2} z({\hat{x}},{\hat{t}})}&= {z_{t}-\varepsilon z_{xx}+l({\hat{x}})z({\hat{x}},{\hat{t}})+m({\hat{x}})z({\hat{x}}-1,{\hat{t}}})\\&= {z_{t}-\varepsilon z_{xx}+\left[ l({\hat{x}})+m({\hat{x}})\right] z({\hat{x}},{\hat{t}})+m({\hat{x}})\left[ z({\hat{x}}-1,{\hat{x}})-z({\hat{x}},{\hat{t}})\right] }\\&={-\varepsilon z_{xx}({\hat{x}},{\hat{t}})+\left[ l({\hat{x}})+m({\hat{x}})\right] z({\hat{x}},{\hat{t}})+m({\hat{x}})\left[ z({\hat{x}}-1,{\hat{t}})-z({\hat{x}},{\hat{t}})\right] }\\&< 0. \end{aligned}$$

The two cases contradict the hypothesis, so that our assumption fails and $$z({\hat{x}},{\hat{t}})\ge 0$$, which implies $$z(x,t)\ge 0$$, $$(x,t)\in {\bar{\Omega }}$$$$\square$$.

### Lemma 2.3

(Stability estimate). The solution of the continuous problem (1) is estimated as $|u(x,t)| \le \mu ^{-1}\Vert g\Vert +\max \left\{ |\alpha (x, t)|, |\beta (2,t)| \right\}$.

### Proof

Let’s define barrier functions as

$$\begin{aligned} \pi ^{\pm }(x,t)=\mu ^{-1}\Vert g\Vert +\max \left\{ |\alpha (x, t)|, |\beta (2,t)| \right\} \pm u(x,t). \end{aligned}$$

Then, we have $\pi ^{\pm }(0,t)\ge 0$ and $\pi ^{\pm }(2,t)\ge 0$.

For $x\in (0,1]$, we get

$$\begin{aligned} L_{\varepsilon ,1}\pi ^{\pm }=\,\pi ^{\pm }_{t}-\varepsilon \pi ^{\pm }_{xx}+l(x)\pi ^{\pm }(x,t) \ge l(x)\max \left\{ |\alpha (0,t)|, |\beta (2,t)| \right\} \ge 0 \end{aligned}$$

For $x\in (1,2)$, we obtain

$$\begin{aligned} L_{\varepsilon ,2}\pi ^{\pm }=\,&\pi ^{\pm }_{t}-\varepsilon \pi ^{\pm }_{xx}+l(x)\pi ^{\pm }(x,t)+m(x)\pi ^{\pm }(x-1,t)\\ \ge&2\mu \max \left\{ |\alpha (0,t)|, |\beta (2,t)| \right\} \ge 0 \end{aligned}$$

Therefore, by Lemma 2.2, the stability estimate holds true. $\square$

### Lemma 2.4

Assuming that Lemmas 2.1 and 2.2 hold true. Then the derivatives of the solution *u*(*x*, *t*) with respect to *t* can be bounded as

$$\begin{aligned} \left| \frac{\partial ^{j}u(x,t)}{\partial t^{j}}\right| \le C, \; (x,t)\in {\bar{\Omega }}, \; j=0, 1, 2. \end{aligned}$$

### Proof

For $j=0$, it implies Lemma 2.1. Let $j=1$. Then on ${\bar{\Omega }}$, we have $u=0$ along the sides $x=0$ and $x=2$, which implies that $u_{t}=0$. On the side $t=0$, we have $u=0$, and hence $u_{xx}=0$. From Eq. (3), we have

10a $$\begin{aligned}&u_{t}(x, 0)-\varepsilon u_{xx}(x, 0)+l(x)u(x,0)=g(x,0)-m(x)\alpha (x-1, 0), \; x\in (0,1], \end{aligned}$$

10b $$\begin{aligned}&u_{t}(x, 0)-\varepsilon u_{xx}(x, 0)+l(x)u(x,0)+m(x)u(x-1, 0)=g(x, 0),\; x\in (1,2). \end{aligned}$$

For $x\in (0, 1]$, $u(x-1,0)=\alpha (x-1, 0)=0$ and for $x\in (1, 2)$, we obtain that $u(x-1,0)=u_0(x-1, 0)=0$. Combining these gives $u(x-1,0)=0$. Then by Eq. (10), we obtain $u_{t}(x, 0)= g(x,0)$. Since *g* is smooth function, it implies that $|u_{t}|\le C$ for sufficiently large C on $\partial \Omega$. Applying the differential operator $L_{\varepsilon }$ on $u_{t}(x,t)$, we obtain $L_{\varepsilon }u_{t}(x,t)=g_{t}(x,t)$, which implies that $|L_{\varepsilon }u_{t}(x,t)|=|g_{t}(x,t)|\le C \; \text {on} \; {\bar{\Omega }}$. Thus, application of Lemma 2.2 gives

$$\begin{aligned} |u_{t}(x,t)|\le C \; \text {on} \; {\bar{\Omega }} \end{aligned}$$

By a similar procedure, for $j=2$ we have $u_{tt}=0$ on the sides $x=0$ and $x=2$, and $u_{xx}=0$ on the side $t=0$. Differentiating Eq. (3) with respect to t, we get

11a $$\begin{aligned}&u_{tt}(x, 0)-\varepsilon u_{xxt}(x, 0)+l(x)u_{t}(x,0)=g_{t}(x,0)-m(x)\alpha _{t}(x-1, 0), \; x\in (0,1], \end{aligned}$$

11b $$\begin{aligned}&u_{tt}(x, 0)-\varepsilon u_{xxt}(x, 0)+l(x)u_{t}(x,0)+m(x)u_{t}(x-1, 0)=g_{t}(x, 0),\; x\in (1,2). \end{aligned}$$

Since $u_{t}(x, 0)= g(x,0)$, we have $u_{xxt}(x, 0)= g_{xx}(x,0)$. And $u(x-1, 0)=0$, implies $u_{t}(x, 0)=0$. Using these results in Eq. (11) yields

12 $$\begin{aligned} u_{tt}(x, 0)=g_{t}(x, 0)+\varepsilon g_{xxt}(x, 0)-l(x)g(x,t). \end{aligned}$$

Since *g* is smooth function, we have $|u_{tt}| \le C$ along the *x*-axis, which implies that $|u_{tt}| \le C$ on $\partial \Omega$. Applying the differential operator on $u_{tt}$, we get $|L_{\varepsilon }u_{tt}(x, t)|\le C$ on $\partial \Omega$. Thus, applying Lemma 2.2 gives

$$\begin{aligned} |u_{tt}(x, t)|\le C \; \text {on} \; {\bar{\Omega }}, \end{aligned}$$

which completes the required proof. $\square$

### Lemma 2.5

The derivatives of the solution *u*(*x*, *t*) with respect to *x* can be bounded as

$$\begin{aligned} \left| \frac{\partial ^{k} u(x, t)}{\partial x^{k}}\right| \le {\left\{ \begin{array}{ll} C(1+\varepsilon ^{-k/2}\delta _{1}(x)), \; 0\le x\le 1,\; 0<t\le T, \\ C(1+\varepsilon ^{-k/2}\delta _{2}(x)), \; 1<x\le 2,\; 0<t\le T, \end{array}\right. } \end{aligned}$$

where $\delta _{1}(x)=\exp (-\sqrt{\frac{\mu }{\varepsilon }}x)+\exp (-\sqrt{\frac{\mu }{\varepsilon }}(1-x))$ and $\delta _{2}(x)=\exp (-\sqrt{\frac{\mu }{\varepsilon }}(x-1))+\exp (-\sqrt{\frac{\mu }{\varepsilon }}(2-x))$ for $k=0, 1, 2, 3.$

### Proof

Consider for $x\in [0, 1].$ For $k=0$, we obtain Lemma 2.1. For $k=1$, fix $t\in [0, T]$ and consider a neighborhood of the form $I=(a, a+\sqrt{\varepsilon })$, $\forall x \in I$. Then, applying the Mean Value Theorem for some $y\in {\bar{I}}$, we get

13 $$\begin{aligned} |u_{x}(y,t)|=\varepsilon ^{-\frac{1}{2}}|u(a+\sqrt{\varepsilon },t)-u(a,t)|\le 2\varepsilon ^{\frac{-1}{2}}\Vert u\Vert . \end{aligned}$$

Now, for any *x* in ${\bar{I}}$, we have

14 $$\begin{aligned} |u_{x}(x,t)|=\, |u_{x}(y,t)+u_{x}(x, t)-u_{x}(y, t)|= |u_{x}(y,t)+\int _{y}^{x}u_{xx}(s, t)ds|. \end{aligned}$$

Using Eq. (1) into Eq. (14) yields

15 $$\begin{aligned} |u_{x}(x,t)|&=\, |u_{x}(y,t)+\frac{1}{\varepsilon }\int _{y}^{x}(u_{t}(s,t)+l(s)u(s,t)+m(s)u(s-1,t)-g(s,t))ds|\nonumber \\&\le |u_{x}(y,t)|+C\varepsilon ^{-1},\; \text {by\; Lemma }\;2.4. \end{aligned}$$

Using Eq. (13) into Eq. (15) gives $|u_{x}(x,t)|\le C\varepsilon ^{\frac{-1}{2}}$. Since $\delta _{1}(x)$ is bounded, we have

$$\begin{aligned} \left| \frac{\partial u(x, t)}{\partial x}\right| \le C(1+\varepsilon ^{-1/2}\delta _{1}(x)), \; 0\le x\le 1,\; 0<t\le T. \end{aligned}$$

Similar procedure holds for $x\in [1, 2]$. Using Eq. (1) and the bounds on *u*(*x*, *t*) and $u_{x}(x,t)$, the bounds for $k=2$ and $k=3$ can be easily obtained. $\square$

### Lemma 2.6

$$\begin{aligned} \left| \frac{\partial ^{2} u(x, t)}{\partial x\partial t}\right| \le {\left\{ \begin{array}{ll} C(1+\varepsilon ^{-1/2}\delta _{1}(x)), \; 0\le x\le 1,\; 0<t\le T, \\ C(1+\varepsilon ^{-1/2}\delta _{2}(x)), \; 1<x\le 2,\; 0<t\le T, \end{array}\right. } \end{aligned}$$

where $$\delta _{1}(x)=\exp (-\sqrt{\frac{\mu }{\varepsilon }}x)+\exp (-\sqrt{\frac{\mu }{\varepsilon }}(1-x))$$ and $$\delta _{2}(x)=\exp (-\sqrt{\frac{\mu }{\varepsilon }}(x-1))+\exp (-\sqrt{\frac{\mu }{\varepsilon }}(2-x))$$.

### Proof

We use the approaches in [17, 18]. $\square$

### Lemma 2.7

$$\begin{aligned} \left| \frac{\partial ^{3} u(x, t)}{\partial x^{2}\partial t}\right| \le {\left\{ \begin{array}{ll} C(1+\varepsilon ^{-1}\delta _{1}(x)), \; 0\le x\le 1,\; 0<t\le T, \\ C(1+\varepsilon ^{-1}\delta _{2}(x)), \; 1<x\le 2,\; 0<t\le T, \end{array}\right. } \end{aligned}$$

where $$\delta _{1}(x)=\exp (-\sqrt{\frac{\mu }{\varepsilon }}x)+\exp (-\sqrt{\frac{\mu }{\varepsilon }}(1-x))$$ and $$\delta _{2}(x)=\exp (-\sqrt{\frac{\mu }{\varepsilon }}(x-1))+\exp (-\sqrt{\frac{\mu }{\varepsilon }}(2-x))$$.

### Proof

We refer the procedures in the proof of Lemma 10 of [19]. $\square$

## Numerical method

### Semi-discretization in the temporal direction

Let’s divide (0, *T*] into equally spaced intervals and form a uniform temporal mesh as $\Omega _{t}^{M}=\{t_{j}=j\Delta t, \ j=0, 1,\ldots , M, \ T=M\Delta t\}$. Then, using implicit Euler method on time derivative, we obtain the semi-discrete scheme as

16 $$\begin{aligned} L_{\varepsilon }^{M}u^{j+1}(x)=\vartheta (x,t_{j+1}), \end{aligned}$$

where

$$\begin{aligned} L_{\varepsilon }^{M}u^{j+1}(x)={\left\{ \begin{array}{ll} -\varepsilon \Delta tu_{xx}^{j+1}+p(x)u^{j+1}(x), \; x\in (0,1],\\ -\varepsilon \Delta tu_{xx}^{j+1}+p(x)u^{j+1}(x)+q(x)u^{j+1}(x-1), \; x\in (1,2) \end{array}\right. } \end{aligned}$$

and

$$\begin{aligned} \vartheta (x,t_{j+1})={\left\{ \begin{array}{ll} \Delta t g(x,t_{j+1})+u^{j}(x)-q(x)\alpha (x-1,t_{j+1}), \; x\in (0, 1], \\ \Delta t g(x,t_{j+1})+u^{j}(x), \; x\in (1, 2) \end{array}\right. } \end{aligned}$$

subject to $u^{j+1}(x)=u_{0}(x),\; x \in {\bar{\Omega }}_{x}$, $u^{j+1}(x)=\alpha (x,t_{j+1}), \; (x,t_{j+1}) \in \Omega _{L}$, $u^{j+1}(2)=\beta (2, t_{j+1}), \; (2,t_{j+1})\in \Omega _{R}$, and for $p(x)=1+\Delta t l(x)$ and $q(x)=\Delta t m(x)$.

#### Lemma 3.1

Let $\psi ^{j+1}(x)$ be a continuous function on ${\bar{\Omega }}_{x}$. If $\psi ^{j+1}(0)\ge 0$, $\psi ^{j+1}(2)\ge 0$ and $L_{\varepsilon } \psi ^{j+1}(x)\ge 0$, $x\in \Omega _{x}$, then $\psi ^{j+1}(x)\ge 0$, $x\in {\bar{\Omega }}_{x}$.

#### Proof

Let $\nu \in [0,2]$ and $\psi ^{j+1}(\nu )=\min _{{\bar{\Omega }}_{x}}\psi ^{j+1}(x)$ and assume that $\psi ^{j+1}(\nu )< 0$. From the given conditions, we have $\nu \notin \partial \Omega _{x}$ and $\psi ^{j+1}_{x}(\nu )=0$, $\psi ^{j+1}_{xx}(\nu )\ge 0$.

**Case 1:** For $\nu \in (0,1]$, we have

$$\begin{aligned} { L^{M}_{\varepsilon ,1} \psi ^{j+1}(\nu )=-\varepsilon \psi ^{j+1}_{xx}(\nu )+p(x)\psi ^{j+1}(\nu )< 0.} \end{aligned}$$

**Case 2:** For $\nu \in (1, 2)$, we have

$$\begin{aligned} { L^{M}_{\varepsilon ,2} \psi ^{j+1}(\nu )}&= {-\varepsilon \psi ^{j+1}_{xx}(\nu )+p(x)\psi ^{j+1}(\nu )+q(\nu )\psi ^{j+1}(\nu -1)}\\&\le {-\varepsilon \psi ^{j+1}_{xx}(\nu )+(p(\nu )+q(\nu ))\psi ^{j+1}(\nu ) < 0.} \end{aligned}$$

By the two cases, the given condition is contradicted, which implies that our assumption is not holds and hence $\psi ^{j+1}(x)\ge 0$, $x\in {\bar{\Omega }}_{x}$. Thus, the maximum principle is satisfied by $L_{\varepsilon , x}^{M}$, and we have

17 $$\begin{aligned} \left\| \left( L_{\varepsilon ,x}^{M}\right) ^{-1}\right\| \le (1+\mu \Delta t)^{-1}, \end{aligned}$$

which is used in estimating the truncation error of the semi-discrete scheme. $\square$

#### Lemma 3.2

The solution $u^{j+1}(x)$ of the semi-discrete problem (16) can be estimated as

$$\begin{aligned} |u^{j+1}(x)|\le \frac{\Vert \vartheta \Vert }{1+\mu \Delta t}+ \max \left\{ |u^{j+1}(0)|, |u^{j+1}(2)| \right\} , \; \forall x\in {\bar{\Omega }}_{x} \end{aligned}$$

#### Proof

Let us define barrier functions as

$$\begin{aligned} { \pi _{\pm }^{j+1}(x)=\frac{\Vert \vartheta \Vert }{1+\mu \Delta t}+ \max \left\{ |u^{j+1}(0)|, |u^{j+1}(2)| \right\} \pm u^{j+1}(x).} \end{aligned}$$

Then, we have $\pi _{\pm }^{j+1}(0)\ge 0$ and $\pi _{\pm }^{j+1}(2)\ge 0$.

For $x\in (0,1]$, we have

$$\begin{aligned} L_{\varepsilon ,1}^{M} \pi _{\pm }^{j+1}(x)&= -\varepsilon (\pi _{\pm })^{j+1}_{xx}+p(x)\pi _{\pm }^{j+1}(x) \\&= \pm \vartheta ^{j+1}(x)+p(x)\frac{\Vert \vartheta \Vert }{1+\mu \Delta t}+p(x) \max \left\{ |u^{j+1}(0)|, |u^{j+1}(2)| \right\} \\&\ge \; \mu \left( \max \left\{ |u^{j+1}(0)|, |u^{j+1}(2)| \right\} \right) \ge \ 0. \end{aligned}$$

For $x\in (1,2)$, we have

$$\begin{aligned} L_{\varepsilon ,2}^{M} \pi _{\pm }^{j+1}(x)&= -\varepsilon (\pi _{\pm })^{j+1}_{xx}+p(x)\pi _{\pm }^{j+1}(x)+q(x)\pi _{\pm }^{j+1}(x-1) \\&= \pm \vartheta ^{j+1}(x)+[p(x)+q(x)]\frac{\Vert \vartheta \Vert }{1+\mu \Delta t} +[p(x) \\&\quad +q(x)] \max \left\{ |u^{j+1}(0)|, |u^{j+1}(2)| \right\} \\&\ge \; \mu \left( \max \left\{ |u^{j+1}(0)|, |u^{j+1}(2)| \right\} \right) \ge 0 \end{aligned}$$

Thus, we obtained that $L_{\varepsilon }^{M}\pi _{\pm }^{j+1}(x)\ge 0$ for all $x\in [0,2]$. Hence, by the semi-discrete maximum principle, the required estimation of $u^{j+1}(x)$ is attained. $\square$

At the $(j+1){th}$ level, we can define the local truncation error $e^{j+1}$ as the difference between the exact solution $u(x,t_{j+1})$ and the approximate solution $u^{j+1}(x)$ of Eq. (16) and the global error estimate $E^{j+1}$ as the contribution of local truncation error up to the $(j+1){th}$ time level.

#### Lemma 3.3

(Local truncation error estimate). Suppose that $\left| u^{(k)}(x,t)\right| \le C$, $(x,t)\in {\bar{\Omega }}$, $k=0, 1, 2$. Then at the $(j+1){th}$ time level, local truncation error is given as $\Vert e^{j+1}\Vert \le C(\Delta t)^{2}$.

#### Proof

We refer Lemma 6 of [20]. $\square$

#### Lemma 3.4

(Estimation of the global error). Suppose that Lemma 3.3 holds. Then the global truncation error is estimated as $\Vert E^{j+1}\Vert \le C(\Delta t)$,  j=0(1)M.

#### Proof

Considering the local truncation error in Lemma 3.3 up to the $(j+1){th}$ time level, we have

$$\begin{aligned} \Vert E^{j+1}\Vert&=\bigg \Vert \sum _{\iota =1}^{j}e^{\iota }\bigg \Vert , \;\; j \le T/\Delta t\\&= \Vert e^{1}+e^{2}+...+e^{j}\Vert \le \Vert e^{1}\Vert +\Vert e^{2}\Vert +...+\Vert e^{j}\Vert \le C(\Delta t), \; j=0(1)M. \end{aligned}$$

Thus, the semi-discrete scheme is convergent of order one in time. $\square$

#### Lemma 3.5

The derivatives of the solution $u^{j+1}(x), \; j+1=1(1)M$ of (16) can be bounded as

$$\begin{aligned} \left| \frac{d^{k}u^{j+1}(x)}{dx^{k}}\right| \le {\left\{ \begin{array}{ll} C\left[ 1+\varepsilon ^{-k/2}\left( \exp (-\sqrt{\frac{\mu }{\varepsilon }}x)+\exp (-\sqrt{\frac{\mu }{\varepsilon }}(1-x))\right) \right] ,\\ \; \; \; \; x\in {{{\bar{\Omega }}}_{x}},\;k=0(1)4, \\ C\left[ 1+\varepsilon ^{-k/2}\left( \exp (-\sqrt{\frac{\mu }{\varepsilon }}(x-1))+\exp (-\sqrt{\frac{\mu }{\varepsilon }}(2-x))\right) \right] ,\\ \; \; \; \; x\in {{{\bar{\Omega }}}_{x}}, k=0(1)4. \end{array}\right. } \end{aligned}$$

#### Proof

See [21]. $$\square$$

### Spatial discretization

Suppose the domain [0, 2] be subdivided into *N* equal intervals of step size *h* and form a uniform mesh as $\Omega ^{N}_{x}=\{0=x_{0},x_{1},\ldots , x_{N/2}=1, x_{N/2+1},\ldots , x_{N}=2, \; x_{i}=ih, i=0(1)N,\; h=2/N\}$.

#### Description and derivation of the tension spline method

On a uniform mesh $\Omega ^{N}_{x}$, a function $S(x,\tau )$ of class $C^{2}[0,2]$ that interpolates *u*(*x*) at $x_{i}$ depends on the compression parameter $\tau$ and reduced to a cubic spline on the interval [0, 2] for $\tau$ approaching to zero is known as parametric cubic spline function [22]. In any interval $[x_{i},x_{i+1}]\subset [0,2]$, the spline function $S(x,\tau )=S(x)$, which satisfies the linear second order differential equation

18 $$\begin{aligned} S_{xx}(x,t_{j+1})-\tau S(x,t_{j+1})&=[S_{xx}(x_{i},t_{j+1})-\tau S(x_{i},t_{j+1})]\left( \frac{x_{i+1}-x}{h}\right) \nonumber \\&\quad +[S_{xx}(x_{i+1},t_{j+1}-\tau S(x_{i+1},t_{j+1})]\left( \frac{x-x_{i}}{h}\right) , \end{aligned}$$

where $S(x_{i}, t_{j+1})=u^{j+1}_{i}$ for $\tau > 0$ is called cubic spline in compression. Solving the homogeneous part of Eq. (18) and setting $\sqrt{\tau }=\frac{\lambda }{h}$ gives

19 $$\begin{aligned} S_{1}(x,t_{j+1})=A\exp \left( \frac{\lambda }{h}(x-x_{i})\right) +B\exp \left( \frac{\lambda }{h}(x_{i+1}-x)\right) , \end{aligned}$$

where *A* and *B* are arbitrary constants. For the non-homogeneous part, let

$$\begin{aligned} { S_{2}(x,t_{j+1})}&= {k\left[ S_{xx}(x_{i},t_{j+1})-\tau S(x_{i},t_{j+1})\right] \left( \frac{x_{i+1}-x}{h}\right) }\\&\quad {+k\left[ S_{xx}(x_{i+1},t_{j+1})-\tau S(x_{i+1},t_{j+1})\right] \left( \frac{x-x_{i}}{h}\right) }. \end{aligned}$$

Substituting in Eq. (18) and simplifying gives $k=-1/\tau$, so that

20 $$\begin{aligned} { S_{2}(x,t_{j+1})}&= {-\left( \frac{h}{\lambda }\right) ^{2}\left[ M_{i}-\left( \frac{\lambda }{h}\right) ^{2} u_{i}^{j+1}\right] \left( \frac{x_{i+1}-x}{h}\right) } \nonumber \\&\quad {-\left( \frac{h}{\lambda }\right) ^{2}\left[ M_{i+1}-\left( \frac{\lambda }{h}\right) ^{2} u_{i+1}^{j+1}\right] \left( \frac{x-x_{i}}{h}\right) ,} \end{aligned}$$

where $M_{i}=S_{xx}(x_{i}, t_{j+1})$ and $M_{i+1}=S_{xx}(x_{i+1}, t_{j+1})$. From (19) and (20) we get

21 $$\begin{aligned} S(x,t_{j+1})&=A\exp \left( \frac{\lambda }{h}(x-x_{i})\right) +B\exp \left( \frac{\lambda }{h}(x_{i+1}-x)\right) -\left( \frac{h}{\lambda }\right) ^{2}\nonumber \\&\quad [M_{i}-\left( \frac{\lambda }{h}\right) ^{2} u_{i}^{j+1}]\left( \frac{x_{i+1}-x}{h}\right) -\left( \frac{h}{\lambda }\right) ^{2}\nonumber \\&\quad [M_{i+1}-\left( \frac{\lambda }{h}\right) ^{2} u_{i+1}^{j+1}]\left( \frac{x-x_{i}}{h}\right) . \end{aligned}$$

The values of the constants *A* and *B* can be determined by the interpolation conditions. That is, in $[x_{i},x_{i+1}]$ from Eq. (21), we obtain

22 $$\begin{aligned} S(x_{i}, t_{j+1})&=A+B\exp (\lambda )-\left( \frac{h}{\lambda }\right) ^{2}\left[ M_{i}-\left( \frac{\lambda }{h}\right) ^{2} u_{i}^{j+1}\right] \end{aligned}$$

and

23 $$\begin{aligned} {S(x_{i+1}, t_{j+1})=A\exp (\lambda )+B-\left( \frac{h}{\lambda }\right) ^{2}\left[ M_{i+1}-\left( \frac{\lambda }{h}\right) ^{2} u_{i+1}^{j+1}\right] }. \end{aligned}$$

From Eqs. (22) and (23), we can obtain that $A=\frac{h^{2}}{2\lambda ^{2}\sinh (\lambda )}[M_{i+1}-e^{\lambda }M_{i}]$ and $B=\frac{h^{2}}{2\lambda ^{2}\sinh (\lambda )}[M_{i}-e^{\lambda }M_{i+1}]$. Thus, Eq. (21) becomes

24 $$\begin{aligned} S(x,t_{j+1})&= \frac{h^{2}}{2\lambda ^{2}\sinh (\lambda )}\left[ M_{i+1}\sinh \left( \frac{\lambda (x-x_{i})}{h}\right) +M_{i}\sinh \left( \frac{\lambda (x_{i+1}-x)}{h}\right) \right] \nonumber \\&\quad -\left[ \frac{h}{\lambda ^{2}}M_{i}-\frac{1}{h} u_{i}^{j+1}\right] (x_{i+1}-x) -\left[ \frac{h}{\lambda ^{2}}M_{i+1}-\frac{1}{h} u_{i+1}^{j+1}\right] (x-x_{i}), \end{aligned}$$

which is the cubic spline in compression on $[x_{i}, x_{i+1}]$, where $M_{i}=S_{xx}(x_{i},t_{j+1})$. The derivative of Eq. (24) at $(x^{+}_{i},t_{j+1})$ is

25 $$\begin{aligned} S_{x}(x^{+}_{i},t_{j+1})=\,\frac{u_{j+1}^{i+1}-u^{j+1}_{i}}{h}-\frac{hM_{i+1}}{\lambda ^{2}}\left( 1-\frac{\lambda }{\sinh (\lambda )}\right) -\frac{hM_{i}}{\lambda ^{2}}\left( \lambda \coth (\lambda )-1\right) . \end{aligned}$$

Similarly for $x\in [x_{i-1}, x_{i}]$, we obtain

26 $$\begin{aligned} S_{x}(x^{-}_{i},t_{j+1})=\,\frac{u_{i}^{j+1}-u^{j+1}_{i-1}}{h}+\frac{hM_{i}\left( \lambda \coth (\lambda )-1\right) }{\lambda ^{2}}+\frac{hM_{i-1}}{\lambda ^{2}}\left( 1-\frac{\lambda }{\sinh (\lambda )}\right) . \end{aligned}$$

From Eqs. (25) and (26) at the mesh point $x_{i}$, we obtain

27 $$\begin{aligned} h^{2}\left( \lambda _{1}M_{i-1}+2\lambda _{2}M_{i}+\lambda _{1}M_{i+1}\right) =u_{i-1}^{j+1}-2u_{i}^{j+1}+u_{i+1}^{j+1}, \end{aligned}$$

where $\lambda _{1}=\frac{1}{\lambda ^{2}}\left( 1-\frac{\lambda }{\sinh (\lambda )}\right)$ and $\lambda _{2}=\frac{1}{\lambda ^{2}}(\lambda \coth (\lambda )-1)$. The consistency condition in Eq. (27) is a guarantee for the continuity of the first derivative of the spline function at the interior points. From the time semi-discrete problem (16), we have

28a $$\begin{aligned}&\varepsilon \Delta tM_{i}=p_{i}u^{j+1}_{i}+q_{i}u^{j+1}_{i-N/2}-\Delta tg^{j+1}_{i}-u^{j}_{i}, \end{aligned}$$

28b $$\begin{aligned}&\varepsilon \Delta tM_{i\pm 1}=p_{i\pm 1}u^{j+1}_{i\pm 1}+q_{i\pm 1}u^{j+1}_{i\pm 1-N/2}-\Delta tg^{j+1}_{i}-u^{j}_{i\pm 1}, \end{aligned}$$

where $p_{i}=1+\Delta tl_{i}$ and $q_{i}=\Delta tm_{i}$. Inserting Eq. (28) into Eq. (27) and rearranging yields

29 $$\begin{aligned}&(-\varepsilon \Delta t+\lambda _{1}h^{2}p_{i-1})u_{i-1}^{j+1}+(2\varepsilon \Delta t+2\lambda _{2}h^{2}p_{i})u_{i}^{j+1}+(-\varepsilon \Delta t+\lambda _{1}h^{2}p_{i+1})u_{i+1}^{j+1} \nonumber \\&\quad =\lambda _{1}h^{2}u_{i-1}^{j}+2\lambda _{2}h^{2}u_{i}^{j}+\lambda _{1}h^{2}u_{i+1}^{j}-\lambda _{1}h^{2}q_{i-1}u^{j+1}(x_{i-1}-1) \nonumber \\&\qquad -2\lambda _{2}h^{2}q_{i}u^{j+1}(x_{i}-1) -\lambda _{1}h^{2}q_{i+1}u^{j+1}(x_{i+1}-1)+\lambda _{1}h^{2}\Delta tg^{j+1}_{i-1}\nonumber \\&\qquad +2\lambda _{2}h^{2}\Delta tg^{j+1}_{i}+\lambda _{1}h^{2}\Delta tg^{j+1}_{i+1}, \; i=1(1)N-1, j=0(1)M-1. \end{aligned}$$

#### Exponential fitting factor

To control the influence of $\varepsilon$ in the region of the layers, we introduce an exponential fitting factor. By analogous procedures in [23], the analytical solution of Eq. (16) is written as

30 $$\begin{aligned} u^{j+1}(x)&=\eta _{1}\exp \left( \sqrt{\frac{p_{i}}{\varepsilon \Delta t}}(x-x_{i})\right) +\eta _{2}\exp \left( -\sqrt{\frac{p_{i}}{\varepsilon \Delta t}}(x-x_{i})\right) \nonumber \\&\quad -\frac{1}{p_{i}}\left[ q_{i}u^{j+1}(x_{i}-1) -\Delta tg(x_{i},t_{j+1})-u^{j}(x_{i})\right] ,\; x\in (x_{i-1}, x_{i+1}), \end{aligned}$$

where the arbitrary constants $\eta _{1}$ and $\eta _{2}$ are determined using the conditions $u^{j+1}(x_{i\pm 1})=u^{j+1}_{i\pm 1}$ and $u^{j+1}(x_{i})=u^{j+1}_{i}$ as

31 $$\begin{aligned} \eta _{1}&=\frac{u_{i-1}^{j+1}-2u_{i}^{j+1}+u_{i+1}^{j+1}}{2(\exp (\rho \sqrt{\frac{p_{i}}{\Delta t}})-2+\exp (-\rho \sqrt{\frac{p_{i}}{\Delta t}}))}+\frac{u_{i-1}^{j+1}-u_{i+1}^{j+1}}{2(\exp (\rho \sqrt{\frac{p_{i}}{\Delta t}})+\exp (-\rho \sqrt{\frac{p_{i}}{\Delta t}}))}, \end{aligned}$$

32 $$\begin{aligned} \eta _{2}&=\frac{u_{i-1}^{j+1}-2u_{i}^{j+1}+u_{i+1}^{j+1}}{2(\exp (\rho \sqrt{\frac{p_{i}}{\Delta t}})-2+\exp (-\rho \sqrt{\frac{p_{i}}{\Delta t}}))}-\frac{u_{i-1}^{j+1}-u_{i+1}^{j+1}}{2(\exp (\rho \sqrt{\frac{p_{i}}{\Delta t}})+\exp (-\rho \sqrt{\frac{p_{i}}{\Delta t}}))}. \end{aligned}$$

Then, introducing a fitting factor $\sigma$ on (0, 1], we obtain

33 $$\begin{gathered} \frac{{\varepsilon \Delta t\sigma }}{{{h^2}}}\left( {u_{i + 1}^{j + 1} - 2u_i^{j + 1} + u_{i - 1}^{j + 1}} \right) - {p_i}{\eta _1}\exp \left( {\sqrt {\frac{{{p_i}}}{{\varepsilon \Delta t}}} (x - {x_i})} \right) \hfill \\ + {p_i}\left[ {{\eta _2}\exp \left( { - \sqrt {\frac{{{p_i}}}{{\varepsilon \Delta t}}} (x - {x_i})} \right) - \frac{1}{{{p_i}}}\left( {{q_i}{u^{j + 1}}({x_i} - 1) - \Delta t{g^{j + 1}}({x_i}) - {u^j}({x_i})} \right)} \right] \hfill \\ - {q_i}{u^{j + 1}}({x_i} - 1) = \Delta t{g^{j + 1}}({x_i}) - u_i^j \hfill \\ \end{gathered}$$

On simplification of Eq. (33) for $i=1, 2,\ldots , N/2$, we obtain the fitting factor

34 $$\begin{aligned} \sigma _{1}=\left( \frac{\rho /2 \sqrt{p(0)/\Delta t}}{\sinh (\rho /2 \sqrt{p(0)/\Delta t})}\right) ^{2}, \end{aligned}$$

where $p(0)=1+\Delta tl(0)$ and $\rho =h/\sqrt{\varepsilon }$. Similarly for $i=N/2+1, N/2+2,\ldots , N$, we obtain the fitting factor as

35 $$\begin{aligned} \sigma _{2}=\left( \frac{\rho /2 \sqrt{p(2)/\Delta t}}{\sinh (\rho /2 \sqrt{p(2)/\Delta t})}\right) ^{2}, \end{aligned}$$

Thus, with the fitting factor $\sigma _{1}$ and $\sigma _{2}$ in Eq. (29), we obtain a fully-discrete numerical scheme as

36 $$\begin{aligned} L^{N, M}_{\varepsilon }u_{i}^{j+1} = \vartheta (x_{i}, t_{j}), \end{aligned}$$

where

$$\begin{aligned} {L^{N, M}_{\varepsilon }u_{i}^{j+1} }=&{{\left\{ \begin{array}{ll} (-\varepsilon \sigma _{1}\Delta t+\lambda _{1}h^{2}p_{i-1})u_{i-1}^{j+1}+(2\varepsilon \sigma _{1}\Delta t+2\lambda _{2}h^{2}p_{i})u_{i}^{j+1}\\ +(-\varepsilon \sigma _{1}\Delta t+\lambda _{1}h^{2}p_{i+1})u_{i+1}^{j+1}, \; i=1(1)N/2,\\ (-\varepsilon \sigma _{2}\Delta t+\lambda _{1}h^{2}p_{i-1})u_{i-1}^{j+1}+(2\varepsilon \sigma _{2}\Delta t+2\lambda _{2}h^{2}p_{i})u_{i}^{j+1} \\ +(-\varepsilon \sigma _{2}\Delta t+\lambda _{1}h^{2}p_{i+1})u_{i+1}^{j+1} +\lambda _{1}h^{2}q_{i-1}u^{j+1}_{i-1-N/2}\\ +2\lambda _{2}h^{2}q_{i}u^{j+1}_{i-N/2} +\lambda _{1}h^{2}q_{i+1}u^{j+1}_{i+1-N/2},\; i=N/2+1(1) N, \end{array}\right. }}\\ \text {and} \\ {\vartheta (x_{i}, t_{j})}=&{{\left\{ \begin{array}{ll} \lambda _{1}h^{2}u_{i-1}^{j}+2\lambda _{2}h^{2}u_{i}^{j}+\lambda _{1}h^{2}u_{i+1}^{j}-\lambda _{1}h^{2}q_{i-1}\alpha ^{j+1}_{i-1-N/2}\\ -2\lambda _{2}h^{2}q_{i}\alpha ^{j+1}_{i-N/2} -\lambda _{1}h^{2}q_{i+1}\alpha ^{j+1}_{i+1-N/2} +\lambda _{1}h^{2}\Delta tg^{j+1}_{i-1}\\ +2\lambda _{2}h^{2}\Delta tg^{j+1}_{i} +\lambda _{1}h^{2}\Delta tg^{j+1}_{i+1}, \; i=1(1)N/2, \\ \lambda _{1}h^{2}u_{i-1}^{j}+2\lambda _{2}h^{2}u_{i}^{j}+\lambda _{1}h^{2}u_{i+1}^{j} +\lambda _{1}h^{2}\Delta tg^{j+1}_{i-1}\\ +2\lambda _{2}h^{2}\Delta tg^{j+1}_{i}+\lambda _{1}h^{2}\Delta tg^{j+1}_{i+1}, \; i=N/2(1)N. \end{array}\right. } } \end{aligned}$$

From Eq. (36), we obtain a system of equation as

37 $$\begin{aligned} \gamma _{1}^{-}u_{i-1}^{j+1}+\gamma _{1}^{0}u_{i}^{j+1}+\gamma _{1}^{+}u_{i+1}^{j+1}=G_{i, j} \end{aligned}$$

with $u^{j+1}_{0}=u^{j+1}(0)$ and $u^{j+1}_{N}=u^{j+1}(x_{N})$, where

$$\begin{aligned} \gamma _{1}^{-}&=-\varepsilon \sigma _{1}\Delta t+\lambda _{1}h^{2}p_{i-1}, \\ \gamma _{1}^{0}&=2\varepsilon \sigma _{1}\Delta t+2\lambda _{2}h^{2}p_{i}, \\ r_{1}^{+}&=-\varepsilon \sigma _{1}\Delta t+\lambda _{1}h^{2}p_{i+1}, \\ G_{i, j}&= {{\left\{ \begin{array}{ll} \lambda _{1}h^{2}u_{i-1}^{j}+2\lambda _{2}h^{2}u_{i}^{j}+\lambda _{1}h^{2}u_{i+1}^{j}-\lambda _{1}h^{2}q_{i-1}\alpha ^{j+1}_{i-1-N/2} \\ -2\lambda _{2}h^{2}q_{i}\alpha ^{j+1}_{i-N/2} -\lambda _{1}h^{2}q_{i+1}\alpha ^{j+1}_{i+1-N/2} +\lambda _{1}h^{2}\Delta tg^{j+1}_{i-1}\\ +2\lambda _{2}h^{2}\Delta tg^{j+1}_{i}+\lambda _{1}h^{2}\Delta tg^{j+1}_{i+1}, \; i=1(1)N/2, \\ \lambda _{1}h^{2}u_{i-1}^{j}+2\lambda _{2}h^{2}u_{i}^{j}+\lambda _{1}h^{2}u_{i+1}^{j}-\lambda _{1}h^{2}q_{i-1}u^{j+1}_{i-1-N/2} \\ -2\lambda _{2}h^{2}q_{i}u^{j+1}_{i-N/2} -\lambda _{1}h^{2}q_{i+1}u^{j+1}_{i+1-N/2} +\lambda _{1}h^{2}\Delta tg^{j+1}_{i-1}\\ +2\lambda _{2}h^{2}\Delta tg^{j+1}_{i}+\lambda _{1}h^{2}\Delta tg^{j+1}_{i+1},\; i=N/2(1)N. \end{array}\right. }} \end{aligned}$$

The systems in Eq. (37) is solved easily using a suitable solver of system of equations.

### Discrete stability and uniform convergence

#### Lemma 3.6

Let $\varsigma \in \{0, 1, 2,\ldots , N\}$ and $\psi ^{j+1}_{\varsigma }=\min _{{\bar{\Omega }}^{N, M}}\psi ^{j+1}_{i}$ and assume that $\psi ^{j+1}_{\varsigma }< 0$. For a mesh function $\psi ^{j+1}_{i}$ if $\psi ^{j+1}_{0}\ge 0$, $\psi ^{j+1}_{N}\ge 0$ and $L^{N, M}_{\varepsilon } \psi ^{j+1}_{\varsigma }\ge 0$, $\varsigma =1, 2,\ldots , N-1$, then $\psi ^{j+1}_{i}\ge 0$, $i=0, 1,\ldots , N$.

#### Proof

For $\varsigma =0(1)N$ and $\psi ^{j+1}_{\varsigma }=\min _{{\bar{\Omega }}^{N,M}}\psi ^{j+1}_{i}$, suppose that $\psi ^{j+1}_{\varsigma }< 0$. From the given condition, it is clear that $\varsigma \notin \{0, N\}$. So, we consider the following two cases.

**Case 1:** When $\varsigma =1(1)N/2$, we have

$$\begin{aligned} { L^{N, M}_{\varepsilon , 1}\psi ^{j+1}_{\varsigma }}&={ (-\varepsilon \sigma _{1}\Delta t+\lambda _{1}h^{2}p_{\varsigma -1})\psi _{\varsigma -1}^{j+1}+(2\varepsilon \sigma _{1}\Delta t+2\lambda _{2}h^{2}p_{\varsigma })\psi _{\varsigma }^{j+1}} \\&\quad { +(-\varepsilon \sigma _{1}\Delta t+\lambda _{1}h^{2}p_{\varsigma +1})\psi _{\varsigma +1}^{j+1}< 0.} \end{aligned}$$

**Case 2:** When $\varsigma =N/2+1(1)N-1$, we have

$$\begin{aligned} { L^{N, M}_{\varepsilon , 2}\psi ^{j+1}_{\varsigma }}&= {(-\varepsilon \sigma _{2}\Delta t+\lambda _{1}h^{2}p_{\varsigma -1})\psi _{\varsigma -1}^{j+1}+(2\varepsilon \sigma _{2}\Delta t+2\lambda _{2}h^{2}p_{\varsigma })\psi _{\varsigma }^{j+1}}\\&\quad {+(-\varepsilon \sigma _{2}\Delta t+\lambda _{1}h^{2}p_{\varsigma +1})\psi _{\varsigma +1}^{j+1}+\lambda _{1}h^{2}q_{\varsigma -1}\psi ^{j+1}_{\varsigma -1-N/2}} \\&\quad {+2\lambda _{2}h^{2}q_{\varsigma }u^{j+1}_{\varsigma -N/2} +\lambda _{1}h^{2}q_{\varsigma +1}\psi ^{j+1}_{\varsigma +1-N/2}}\\&\le { (-\varepsilon \sigma _{2}\Delta t+\lambda _{1}h^{2}p_{\varsigma -1})\psi _{\varsigma -1}^{j+1}+(2\varepsilon \sigma _{2}\Delta t+2\lambda _{2}h^{2}p_{\varsigma })\psi _{\varsigma }^{j+1}}\\&\quad {+(-\varepsilon \sigma _{2}\Delta t+\lambda _{1}h^{2}p_{\varsigma +1})\psi _{\varsigma +1}^{j+1}+\lambda _{1}h^{2}q_{\varsigma -1}\psi ^{j+1}_{\varsigma -1}+2\lambda _{2}h^{2}q_{\varsigma }u^{j+1}_{\varsigma }}\\&\quad {+\lambda _{1}h^{2}q_{\varsigma +1}\psi ^{j+1}_{\varsigma +1} < 0.} \end{aligned}$$

From the two cases, we see that $L^{N, M}_{\varepsilon }\psi ^{j+1}_{\varsigma } < 0$, which contradicts the given hypothesis. Thus, our assumption fails, and hence $\psi ^{j+1}_{i}\ge 0$, $i=0(1)N$. $\square$

#### Lemma 3.7

The solution $u^{j+1}_{i}$ of the difference scheme in Eq. (36) is estimated as $|u^{j+1}_{i}|\le (1+\mu \Delta t)^{-1}\Vert \vartheta \Vert + \max \left\{ |u^{j+1}_{0}|, |u^{j+1}_{N}| \right\} , \; \forall i=0, 1,\ldots , N$.

#### Proof

Let $\pi _{i, \pm }^{j+1}$ be barrier functions defined by

$$\begin{aligned} \pi _{i, \pm }^{j+1}=(1+\mu \Delta t)^{-1}\Vert \vartheta \Vert + \max \left\{ |u^{j+1}_{0}|, |u^{j+1}_{N}| \right\} \pm u^{j+1}_{i} \end{aligned}$$

Then, we have $\pi _{0,\pm }^{j+1}\ge 0$ and $\pi _{N, \pm }^{j+1}\ge 0$. Now, let $\omega = (1+\mu \Delta t)^{-1}\Vert \vartheta \Vert + \max \left\{ |u^{j+1}_{0}|, |u^{j+1}_{N}| \right\}$. Then, when $i=1,2,\ldots , N/2$, we have

$$\begin{aligned} L_{\varepsilon ,1}^{N, M} \pi _{i, \pm }^{j+1}&=(-\varepsilon \sigma _{1}\Delta t+\lambda _{1}h^{2}p_{i-1})(\omega \pm u^{j+1}_{i-1})+(2\varepsilon \sigma _{1}\Delta t+2\lambda _{2}h^{2}p_{i})(\omega \pm u^{j+1}_{i})\\&\quad +(-\varepsilon \sigma _{1}\Delta t+\lambda _{1}h^{2}p_{i+1})(\omega \pm u^{j+1}_{i+1})\\&\ge (\lambda _{1}h^{2}p_{i-1}+2\lambda _{2}h^{2}p_{i}+\lambda _{1}h^{2}p_{i+1})\left[ \max \{|u^{j+1}_{0}|, |u^{j+1}_{N}| \}\right] \ge 0. \end{aligned}$$

And for $i=N/2+1, N/2+2,\ldots , N-1$, we have

$$\begin{aligned} L_{\varepsilon , 2}^{N, M} \pi _{i, \pm }^{j+1}&=(-\varepsilon \sigma _{2}\Delta t+\lambda _{1}h^{2}p_{i-1})(\omega \pm u^{j+1}_{i-1})+(2\varepsilon \sigma _{2}\Delta t+2\lambda _{2}h^{2}p_{i})(\omega \pm u^{j+1}_{i})\\&\quad +(-\varepsilon \sigma _{2}\Delta t+\lambda _{1}h^{2}p_{i+1})(\omega \pm u^{j+1}_{i+1}) +\lambda _{1}h^{2}q_{i-1}(\omega +u^{j+1}_{i-1-N/2})\\&\quad +2\lambda _{2}h^{2}q_{i}(\omega \pm u^{j+1}_{i-N/2}) +\lambda _{1}h^{2}q_{i+1}(\omega \pm u^{j+1}_{i+1-N/2})\\&= h^{2}(\lambda _{1}p_{i-1}+2\lambda _{2}p_{i}+\lambda _{1}p_{i+1}+\lambda _{1}q_{i-1}+2\lambda _{1}q_{i}+\lambda _{1}q_{i+1})\\&\quad \left[ (1+\mu \Delta t)^{-1}\Vert \vartheta \Vert + \max \{|u^{j+1}_{0}|, |u^{j+1}_{N}| \}\right] \pm \vartheta (x_{i}, t_{j})\\&\ge h^{2}[\lambda _{1}(p_{i-1}+p_{i+1}+q_{i-1}+q_{i+1})\\&\quad +2\lambda _{2}(p_{i}+q_{i})]\left[ \max \{|u^{j+1}_{0}|, |u^{j+1}_{N}| \}\right] \ge 0. \end{aligned}$$

Therefore, we have $L_{\varepsilon }^{M}\pi _{i, \pm }^{j+1}\ge 0$, $i=0, 1, 2,\ldots , N$, and applying Lemma 3.6, the required stability estimate of $u^{j+1}_{i}$ is implied. $\square$

#### Theorem 3.1

Let $u^{j+1}(x_{i})$ and $u^{j+1}_{i}$ be the solutions of the schemes (16) and (36), respectively. Then, the error estimate in the spatial discretization is given by

$$\begin{aligned} |u^{j+1}(x_{i})-u^{j+1}_{i}|\le CN^{-2}, \; i=0, 1, 2,\ldots , N. \end{aligned}$$

#### Proof

For $i=0, 1,\ldots , N/2$, the truncation error is

$$\begin{aligned} |L^{M}_{\varepsilon }u^{j+1}(x_{i})-L^{N, M}_{\varepsilon }u^{j+1}_{i}|&=|-\varepsilon \Delta t u^{j+1}_{xx}+p(x_{i})u^{j+1}(x_{i})+\varepsilon \sigma _{1}\Delta t\delta ^{2}_{x}u^{j+1}_{i}\\&\quad -\lambda _{1}p_{i+1}u^{j+1}_{i+1}-2\lambda _{2}p_{i}u^{j+1}_{i}-\lambda _{1}p_{i-1}u^{j+1}_{i-1}| \end{aligned}$$

Using Taylor’s series expansion for $u^{j+1}_{i\pm 1}$, we obtain

38 $$\begin{aligned} |L^{M}_{\varepsilon }u^{j+1}(x_{i})-L^{N, M}_{\varepsilon }u^{j+1}_{i}|&=|-\varepsilon \Delta t u^{j+1}_{xx}+p(x_{i})u^{j+1}(x_{i})+\varepsilon \sigma _{1}\Delta t(u^{j+1}_{xx} \nonumber \\&\quad +\frac{h^{2}}{12}u^{j+1}_{xxxx} +\frac{h^{4}}{360}u^{j+1}_{xxxxxx}+O(h^{6})) -\lambda _{1}p_{i+1}(u^{j+1}_{i} \nonumber \\&\quad +hu^{j+1}_{x}+\frac{h^{2}}{2}u^{j+1}_{xx} +\frac{h^{3}}{6}u^{j+1}_{xxx}+\frac{h^{4}}{24}u^{j+1}_{xxxx}\nonumber \\&\quad +\frac{h^{5}}{120}u^{j+1}_{xxxxx} +O(h^{6}))-2\lambda _{2}p_{i}u^{j+1}_{i} \nonumber \\&\quad -\lambda _{1}p_{i-1}(u^{j+1}_{i}-hu^{j+1}_{x}+\frac{h^{2}}{2}u^{j+1}_{xx}-\frac{h^{3}}{6}u^{j+1}_{xxx} \nonumber \\&\quad +\frac{h^{4}}{24}u^{j+1}_{xxxx} -\frac{h^{5}}{120}u^{j+1}_{xxxxx}+O(h^{6}))| \end{aligned}$$

For $\lambda _{1}$ and $\lambda _{2}$ satisfying $2\lambda _{2}=1-2\lambda _{1}$, and using Taylor’s series expansion on $p_{i\pm 1}$ and $\sigma _{1}$, after certain manipulation Eq. (38) becomes

$$\begin{aligned} |L^{M}_{\varepsilon }u^{j+1}(x_{i})-L^{N, M}_{\varepsilon }u^{j+1}_{i}|&=|(\frac{\varepsilon \Delta t}{12}u^{j+1}_{xxxx}-\lambda _{1} p_{i}u^{j+1}_{xx} )h^{2}+(\frac{\varepsilon \Delta t}{360}u^{j+1}_{xxxxxx}\\&\quad -\frac{\Delta tp_{i}}{144}u^{j+1}_{xxxx} -\frac{\lambda _{1}p_{i}}{24}u^{j+1}_{xxxx})h^{4}+O(h^{6})|\\&\le |\frac{\varepsilon \Delta t}{12}u^{j+1}_{xxxx}-\lambda _{1} p_{i}u^{j+1}_{xx} |h^{2}+|\frac{\varepsilon \Delta t}{360}u^{j+1}_{xxxxxx}\\&\quad -\frac{\Delta tp_{i}}{144}u^{j+1}_{xxxx} -\frac{\lambda _{1}p_{i}}{24}u^{j+1}_{xxxx}|h^{4}+O(h^{6}) \le Ch^{2}. \end{aligned}$$

Now, invoking Lemma 3.6 yields

39 $$\begin{aligned} |u^{j+1}(x_{i})-u^{j+1}_{i}|\le Ch^{2}, \; i=0, 1, 2,\ldots , N/2. \end{aligned}$$

For $i=\frac{N}{2}+1, \frac{N}{2}+2,\ldots , N$, we have

40 $$\begin{aligned} |L^{M}_{\varepsilon }u^{j+1}(x_{i})-L^{N, M}_{\varepsilon }u^{j+1}_{i}|&=|-\varepsilon \Delta t u^{j+1}_{xx}+p(x_{i})u^{j+1}(x_{i})+q(x_{i})u^{j+1}(x_{i-\frac{N}{2}}) \nonumber \\&\quad +\varepsilon \sigma _{2}\Delta t\delta ^{2}_{x}u^{j+1}_{i}-\lambda _{1}p_{i+1}u^{j+1}_{i+1}-2\lambda _{2}p_{i}u^{j+1}_{i} \nonumber \\&\quad -\lambda _{1}p_{i-1}u^{j+1}_{i-1}-\lambda _{1}q_{i-1}u^{j+1}_{i-1-\frac{N}{2}}-2\lambda _{2}q_{i}u^{j+1}_{i-\frac{N}{2}} \nonumber \\&\quad -\lambda _{1}q_{i+1}u^{j+1}_{i+1-\frac{N}{2}}|. \end{aligned}$$

Using Taylor’s series expansion on $u^{j+1}_{i\pm 1}$, $p_{i\pm 1}$, $q_{i\pm 1-\frac{N}{2}}$ and $\sigma _{2}$ in Eq. (40) gives

$$\begin{aligned} |L^{M}_{\varepsilon }u^{j+1}(x_{i})-L^{N, M}_{\varepsilon }u^{j+1}_{i}|&=|(\frac{\varepsilon \Delta t}{12}u^{j+1}_{xxxx}-\lambda _{1} p_{i}u^{j+1}_{xx}-\lambda _{1}q_{i}u^{j+1}_{xx}(x_{i-\frac{N}{2}})\\&\quad -2\lambda _{1}q'_{i}u^{j+1}_{x}(x_{i-\frac{N}{2}})-\lambda _{1}q''_{i}u^{j+1}(x_{i-\frac{N}{2}}) )h^{2} \\&\quad +(\frac{\varepsilon \Delta t}{360}u^{j+1}_{xxxxxx} -\frac{\Delta tp_{i}}{144}u^{j+1}_{xxxx} -\frac{\lambda _{1}p_{i}}{24}u^{j+1}_{xxxx}\\&\quad -\frac{\lambda _{1}q'_{i}}{3}u^{j+1}_{xxx}(x_{i-\frac{N}{2}})-\frac{\lambda _{1}q_{i}}{12}u^{j+1}_{xxxx}(x_{i-\frac{N}{2}}))h^{4}\\&\quad +O(h^{6})|\\&\le |\frac{\varepsilon \Delta t}{12}u^{j+1}_{xxxx}-\lambda _{1} p_{i}u^{j+1}_{xx}-\lambda _{1}q_{i}u^{j+1}_{xx}(x_{i-\frac{N}{2}})\\&\quad -2\lambda _{1}q'_{i}u^{j+1}_{x}(x_{i-\frac{N}{2}})-\lambda _{1}q''_{i}u^{j+1}(x_{i-\frac{N}{2}})|h^{2} \\&\quad +|\frac{\varepsilon \Delta t}{360}u^{j+1}_{xxxxxx} -\frac{\Delta tp_{i}}{144}u^{j+1}_{xxxx} -\frac{\lambda _{1}p_{i}}{24}u^{j+1}_{xxxx}\\&\quad -\frac{\lambda _{1}q'_{i}}{3}u^{j+1}_{xxx}(x_{i-\frac{N}{2}})-\frac{\lambda _{1}q_{i}}{12}u^{j+1}_{xxxx}(x_{i-\frac{N}{2}})|h^{4}\\&\quad +O(h^{6})\\&\le \ Ch^{2}. \end{aligned}$$

Invoking Lemma 3.6 gives

41 $$\begin{aligned} { |u^{j+1}(x_{i})-u^{j+1}_{i}|\le Ch^{2},\; i=\frac{N}{2}+1, \frac{N}{2}+2,\ldots , N.} \end{aligned}$$

Since $h= \frac{2}{N}$, combining the inequalities (39) and (41) gives the required error estimate. Hence, the proposed scheme is uniformly convergent of order two in space. $\square$

#### Theorem 3.2

Let *u*(*x*) be the solution of Eq.(1) and $u^{j+1}_{i}$ be the solution of Eq. (36). Then, the uniform error is estimated as

$$\begin{aligned} \sup \limits _{i=0(1)N, j=0(1) M}|u(x_{i}, t_{j+1})-u^{j+1}_{i}| \le C(\Delta t+N^{-2}) \end{aligned}$$

#### Proof

Combining the proofs of Lemma 3.4 and Theorem 3.1, we can obtain the required uniform error estimate. $\square$

## Numerical experiments, results and discussions

To illustrate the implementation of the present numerical scheme, we solved model problems. Since the exact solutions of both problems are not known, we apply the double mesh principle [24] to determine the maximum nodal error as $E^{N,M}_{\varepsilon }=\max \limits _{1\le i\le N}(u^{N,M}_{i}-u^{2N,2\,M}_{i})$, where $u^{2N,2\,M}(x_{i},t_{j})$ is obtained by doubling the mesh numbers for a fixed transition parameter. The parameter-uniform maximum error is determined as $E^{N,M}=\max \limits _{\varepsilon } E^{N,M}_{\varepsilon }$. The maximum convergence rate of the method is computed as $R_{\varepsilon }^{N,M}=\frac{\log (E_{\varepsilon }^{N,M}/E_{\varepsilon }^{2N,2\,M})}{\log (2)}$ and its uniform convergence rate is determined by $R^{N,M}=\max \limits _{\varepsilon }R_{\varepsilon }^{N,M}$.

### Example 4.1

[10]. Consider $-\frac{\partial u}{dt}+\varepsilon \frac{\partial ^{2}u}{\partial x^{2}}-5u(x,t)+2u(x-1,t)=-2$, subject to $u(x,0)=\sin (\pi x)$, $x\in [0,2]$, $u(x,t)=0$, $(x,t)\in \{(x,t): x\in [-1, 0] \; \text {and}\; t\in [0,2]\}$ and $u(2,t)=0$, $(2,t)\in \{(2,t): 0\le t\le 2\}$.

### Example 4.2

[11]. Consider $-\frac{\partial u}{dt}+\varepsilon \frac{\partial ^{2}u}{\partial x^{2}}-(x+6)u(x,t)+(x^{2}+1)u(x-1,t)=-3$, subject to $u(x,0)=0$, $x\in [0,2]$, $u(x,t)=0$, $(x,t)\in \{(x,t): x\in [-1,0];\; \; t\in [0,2] \}$ and $u(2,t)=0$, $(2,t)\in \{(2,t): t\in [0,2] \}$.

The numerical solutions and error analysis of both examples are computed applying the proposed numerical scheme by using the MATLAB R2019a packages. We computed the examples for $\lambda _{1}=1/24$ and $\lambda _{1}=11/24$. The maximum nodal error and convergence rate of both examples are computed and the results are as given in Tables 1 and 2, respectively. From these tables, we observe that by increasing the number of meshes, the maximum error decreases, while decreasing the value of $\varepsilon$ yields an stabled maximum error. This confirms the uniform convergence of the proposed numerical scheme. Table 3 shows the accuracy of our scheme as compared to other works in the literature.

**Fig. 1:**
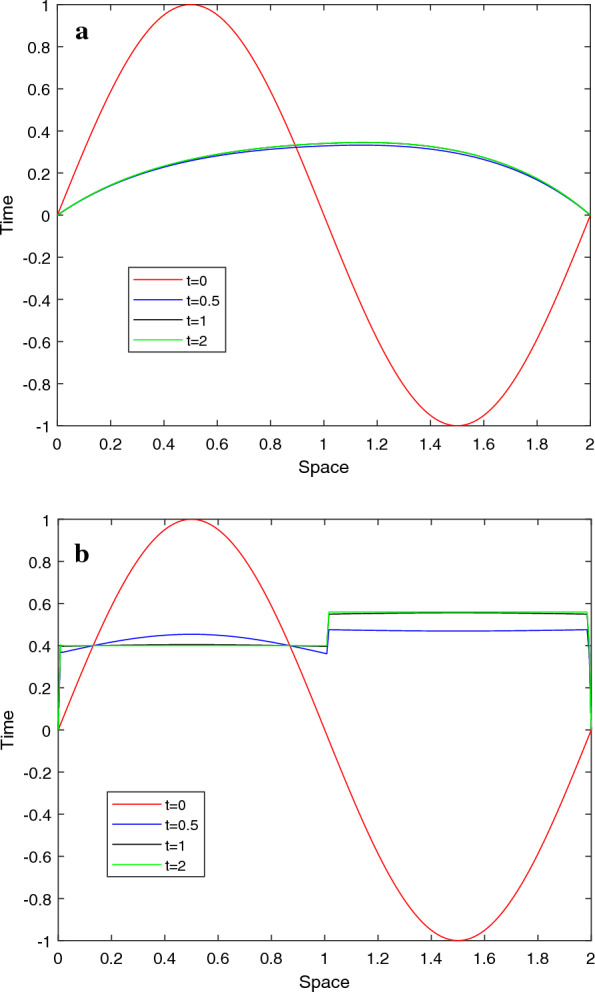
Line plots of the solution for Example 4.1 for $N=128$ at four time levels **a**. $\varepsilon =2^{0}$ and **b**. $\varepsilon =2^{-16}$

**Fig. 2:**
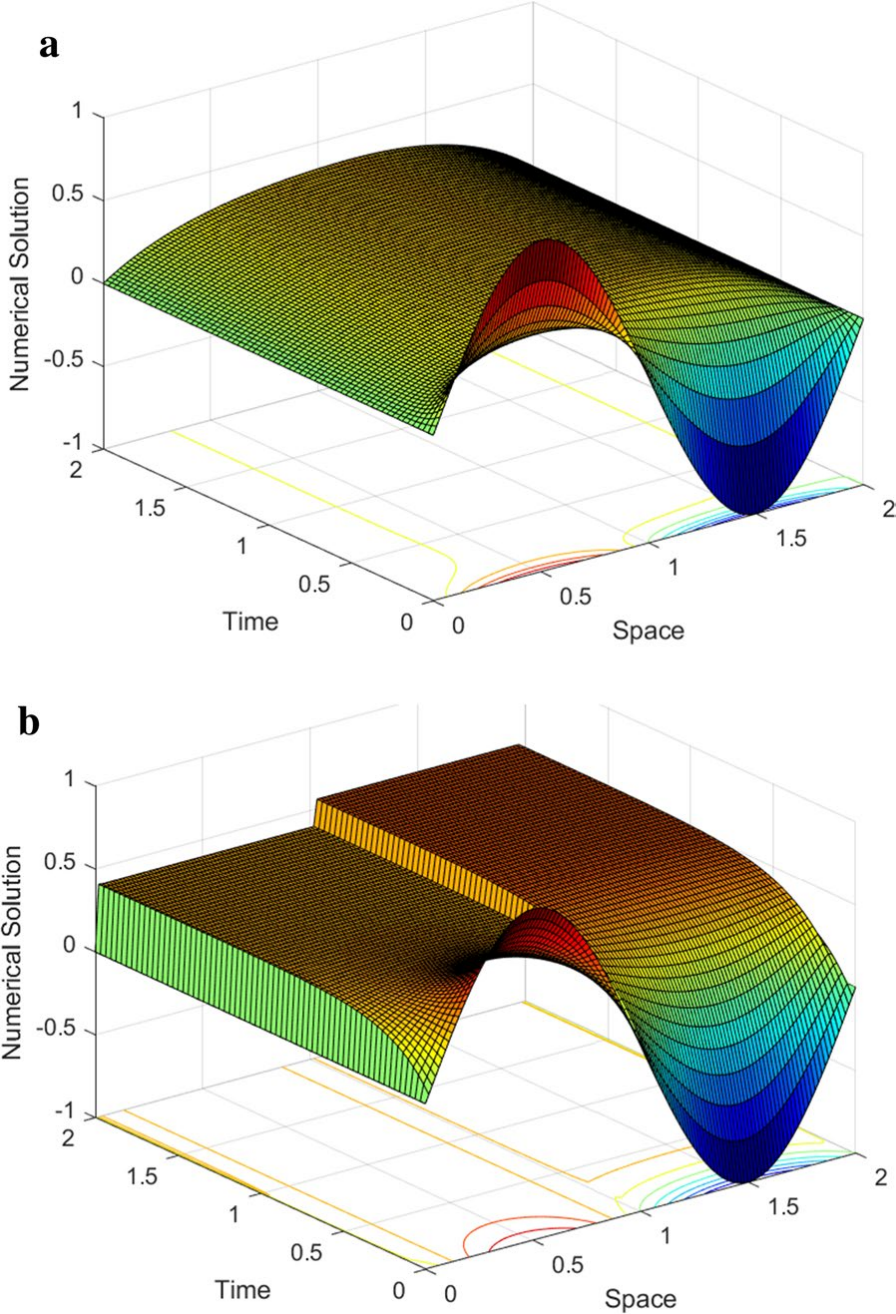
Surface plots of the solution for Example 4.1 for $N=128$ and $M=64$ **a**. $\varepsilon =2^{0}$ and **b**. $\varepsilon =2^{-16}$

**Fig. 3:**
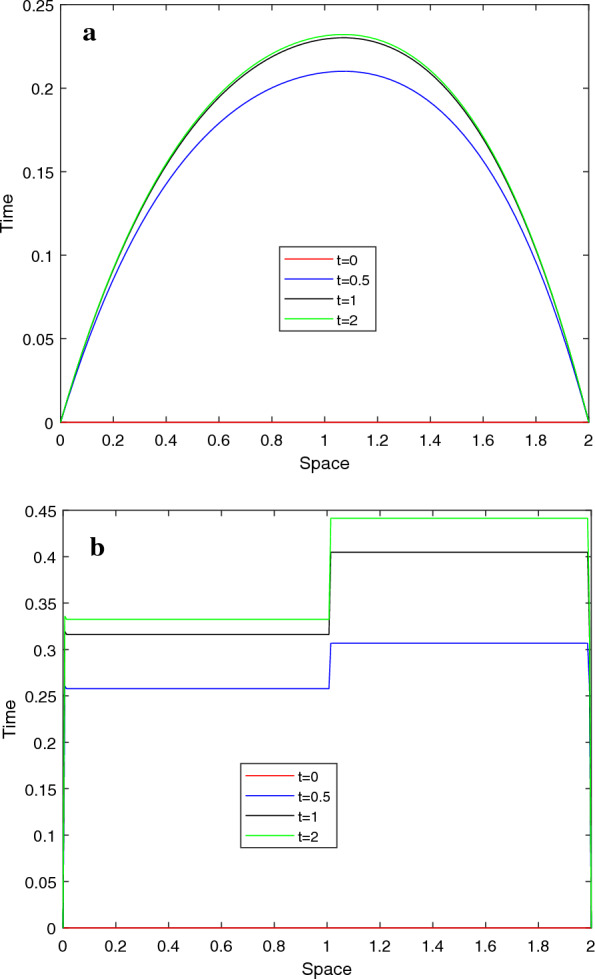
Line plots of the solution for Example 4.2 for $N=144$ at four time levels **a**. $\varepsilon =2^{0}$ and **b**. $\varepsilon =2^{-14}$

**Fig. 4:**
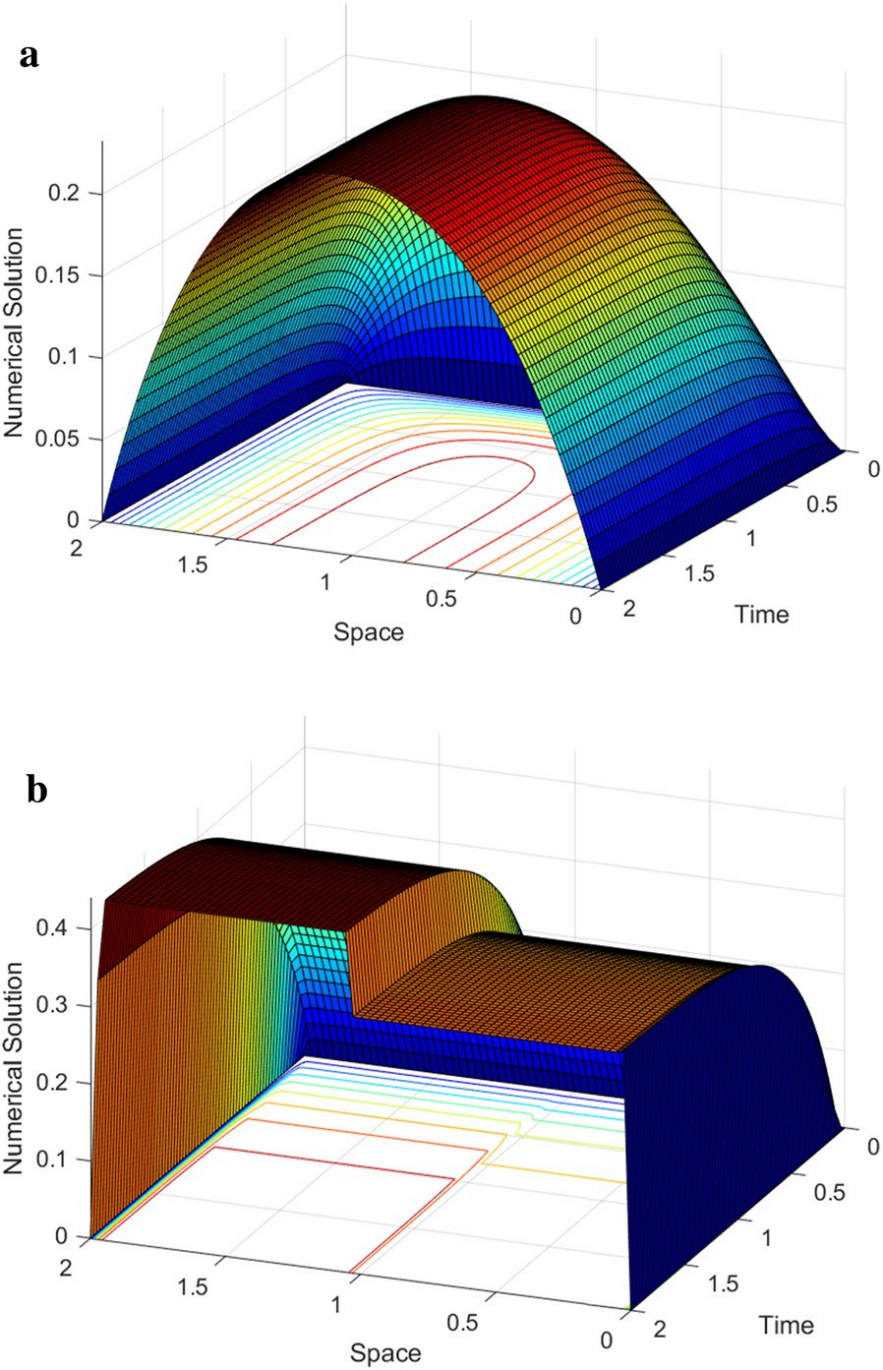
Surface plots of the solution for Example 4.2 for $N=144$ and $M=144$ **a**. $\varepsilon =2^{0}$ and **b**. $\varepsilon =2^{-14}$

**Fig. 5:**
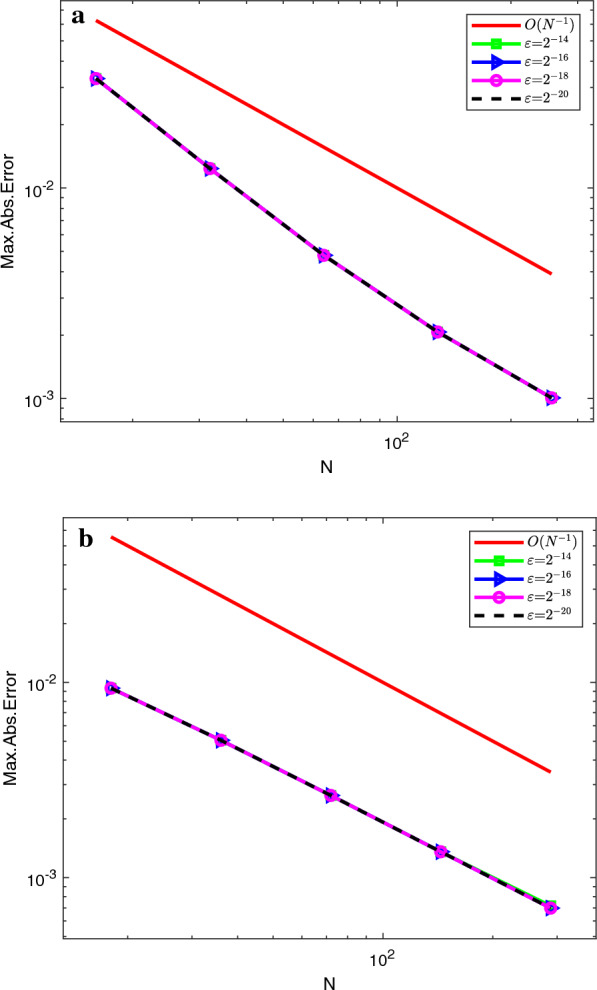
The log-log plots of the Maximum absolute errors with the mesh numbers for Example 4.1 in (**a**) and for Example 4.2 in (**b**)

Graphical simulations of the solutions of the two examples are shown in Figs. 1, 2, 3, 4. From the line plots in Figs. 1 and 3, we observe the solution behaviors at different time levels and $\varepsilon$. Also, to depict the physical behavior of the solutions surface plots are shown in Figs. 2 and 4 for the two examples, respectively. From these figures, we see that as the value of $\varepsilon$ decreases, the width of the layers decreases. Figure 5 shows the log-log plots of the maximum error versus the number of meshes for both examples, which indicates that the developed numerical method is convergent independent of the perturbation parameter.

**Table 1:** $E^{N,M}_{\varepsilon }$, $E^{N,M}$, $R^{N,M}_{\varepsilon }$ and $R^{N,M}$ of Example 4.1

$\varepsilon$	*N* : 16	32	64	128	256
	*M* : 32	64	128	256	512
$2^{-00}$	5.2038e−02	1.7483e−02	4.9785e−03	1.3260e−03	3.4229e−04
	1.5736	1.8122	1.9086	1.9538	
$2^{-02}$	4.7981e−02	1.5766e−02	4.3204e−03	1.1229e−03	2.8612e−04
	1.6056	1.8676	1.9439	1.9725	
$2^{-04}$	4.5798e−02	1.8492e−02	5.3904e−03	1.4174e−03	3.6547e−04
	1.3084	1.7784	1.9271	1.9554	
$2^{-06}$	3.5906e−02	1.8311e−02	6.1997e−03	1.3990e−03	4.5341e−04
	0.9715	1.5624	2.1478	1.6255	
$2^{-08}$	3.3221e−02	1.2981e−02	6.1191e−03	2.7049e−03	1.0428e−03
	1.3557	1.0850	1.1777	1.3751	
$2^{-10}$	3.3117e−02	1.2380e−02	4.8776e−03	2.0203e−03	1.0245e−03
	1.4196	1.3438	1.2716	0.9796	
$2^{-12}$	3.3117e−02	1.2376e−02	4.7903e−03	2.0772e−03	1.0013e−03
	1.4200	1.3694	1.2055	1.0528	
$2^{-14}$	3.3117e−02	1.2376e−02	4.7902e−03	2.0709e−03	1.0076e−03
	1.4200	1.3694	1.2098	1.0393	
$2^{-16}$	3.3117e−02	1.2376e−02	4.7902e−03	2.0709e−03	1.0075e−03
	1.4200	1.3694	1.2098	1.0395	
$2^{-18}$	3.3117e−02	1.2376e−02	4.7902e−03	2.0709e−03	1.0075e−03
	1.4200	1.3694	1.2098	1.0395	
$E^{N,M}$	5.2038e−02	1.8492e−02	6.1997e−03	2.7049e−03	1.0428e−03
$R^{N,M}$	1.4927	1.5766	1.1966	1.3751

**Table 2:** $E^{N,M}_{\varepsilon }$, $E^{N,M}$, $R^{N,M}_{\varepsilon }$ and $R^{N,M}$ of Example 4.2

$\varepsilon$	$N\rightarrow$ 18	36	72	144	288
	$M\rightarrow$ 18	36	72	144	288
$2^{-00}$	4.9315e−03	1.6091e−03	5.5532e−04	1.9610e−04	3.9612e−05
	1.6158	1.5349	1.5017	1.4942	
$2^{-02}$	8.3785e−03	2.7112e−03	9.6535e−04	3.6187e−04	1.3305e−04
	1.6278	1.4898	1.4156	1.4435	
$2^{-04}$	1.2009e−02	4.7851e−03	1.5672e−03	5.6198e−04	2.2895e−04
	1.3275	1.6104	1.4796	1.2955	
$2^{-06}$	1.2133e−02	6.2859e−03	1.9900e−03	7.4889e−04	3.2170e−04
	0.9487	1.6594	1.4099	1.2190	
$2^{-08}$	9.5475e−03	6.7757e−03	2.8381e−03	1.5221e−03	7.7066e−04
	0.4948	1.2554	0.8989	0.9809	
$2^{-10}$	9.3401e−03	5.0933e−03	3.4973e−03	1.6184e−03	7.2280e−04
	0.8748	0.5424	1.1117	1.1629	
$2^{-12}$	9.3397e−03	5.0529e−03	2.6405e−03	1.5946e−03	7.2447e−04
	0.8863	0.9363	0.7276	1.1382	
$2^{-14}$	9.3397e−03	5.0529e−03	2.6371e−03	1.3578e−03	7.1757e−04
	0.8863	0.9382	0.9577	0.9201	
$2^{-16}$	9.3397e−03	5.0529e−03	2.6371e−03	1.3577e−03	6.9879e−04
	0.8863	0.9382	0.9578	0.9582	
$2^{-18}$	9.3397e−03	5.0529e−03	2.6371e−03	1.3577e−03	6.9879e−04
	0.8863	0.9382	0.9578	0.9582	
$E^{N,M}$	1.2133e−02	6.7757e−03	3.4973e−03	1.5946e−03	7.7066e−04
$R^{N,M}$	0.8405	0.9541	1.1330	1.0490

**Table 3:** Comparison of the proposed method and other results in literature

$R_{\varepsilon }^{2N,4M}$ of Example 4.1					
*N* :	64	128	256	512	
*M* :	32	128	512	2048	
Proposed method					
$\varepsilon ^{-16}$	1.8871	2.1168	2.0830	1.9190	
$\varepsilon ^{-18}$	1.8871	2.1168	2.0830	1.9190	
$\varepsilon ^{-20}$	1.8871	2.1168	2.0830	1.9190	
Results in [10]					
$\varepsilon ^{-16}$	1.7908	1.8314	1.5121	1.6261	
$\varepsilon ^{-18}$	1.7908	1.8354	1.5091	1.6257	
$\varepsilon ^{-20}$	1.7908	1.8354	1.5091	1.6257	

$E^{N,M}$ and $R^{N,M}$ of Example 4.2 for $T=1$					
$N=M$ :	18	36	72	144	288
Proposed method					
$E^{N}$	6.7757e−03	3.4973e−03	1.5994e−03	7.1757e−04	3.6436e−04
$R^{N}$	0.9541	1.1287	1.1563	0.9778	
Results [11]					
$E^{N,M}$	1.1200e−02	7.0100-03	2.9700e−03	1.1400e−03	4.0600e−04
$R^{N,M}$	0.6760	1.2390	1.3814	1.4895	

## Conclusion

In this paper, we considered a time dependent singularly perturbed parabolic reaction-diffusion problem involving spatial delay. The influence of the perturbation parameter forms strong boundary layers in the solution and the large delay term gives rise to strong layer at $x=1$. We treated such problem by developing a numerical scheme applying the implicit Euler method in the temporal variable and fitted spline tension method in the spatial variable. The stability estimate and the uniform error bound are investigated and proved. To validate the theoretical findings, we solved two numerical examples. Based on the theoretical and experimental results, we concluded that the proposed numerical scheme is uniformly convergent.

## Data Availability

There is no additional data used for this study.
